# Photocurrent Measurement of PC and PV HgCdTe Detectors

**DOI:** 10.6028/jres.106.024

**Published:** 2001-06-01

**Authors:** George P. Eppeldauer, Robert J. Martin

**Affiliations:** National Institute of Standards and Technology, Gaithersburg, MD 20899-8141; Analog/Digital Integrated Circuits, Inc., Longwood, FL 32750

**Keywords:** detector, drift, HgCdTe, infrared, noise, photoconductive, photocurrent, photodiode, preamplifier

## Abstract

Novel preamplifiers for working standard photoconductive (PC) and photovoltaic (PV) HgCdTe detectors have been developed to maintain the spectral responsivity scale of the National Institute of Standards and Technology (NIST) in the wavelength range of 5 μm to 20 μm. The linear PC mode preamplifier does not need any compensating source to zero the effect of the detector bias current for the preamplifier output. The impedance multiplication concept with a positive feedback buffer amplifier was analyzed and utilized in a bootstrap PV transimpedance amplifier to measure photocurrent of a 200 Ω shunt resistance photodiode with a maximum signal gain of 10^8^ V/A. In spite of the high performance lock-in used as a second-stage signal-amplifier, the signal-to-noise ratio had to be optimized for the output of the photocurrent preamplifiers. Noise and drift were equalized for the output of the PV mode preamplifier. The signal gain errors were calculated to determine the signal frequency range where photocurrent-to-voltage conversion can be performed with very low uncertainties. For the design of both PC and PV detector preamplifiers, the most important gain equations are described. Measurement results on signal ranges and noise performance are discussed.

## 1. Introduction

HgCdTe detectors are widely used for low-level optical radiation measurements in the infrared (IR) range. Working standard HgCdTe radiometers have been developed and calibrated against a transfer standard cryogenic bolometer [1]. The working standards hold the IR spectral responsivity scale and test detectors are calibrated against them. The expectation from the working standards is to propagate and maintain the 0.8 % (coverage factor *k* = 1) standard relative uncertainty of the IR spectral responsivity scale realized on the cryogenic bolometer, with the smallest possible uncertainty increase. One of the most important requirements for working standards development was to produce linear operation in a wide radiant power range. Linear response can be obtained from both photovoltaic (PV) and photoconductive (PC) type devices if the detector short circuit current is measured [2]. HgCdTe detectors have low resistance-area (RA) product resulting in small detector-resistance and high gain for the preamplifier input voltage-noise, even for small area detectors. It is an important design consideration to produce high sensitivity by keeping the voltage-noise gain low. Also, the spatial response non-uniformity of HgCdTe detectors is poor because of the difficulties associated with producing homogeneous material. For accurate measurements, it is necessary to overfill these detectors with a uniform field of radiation to average out high response non-uniformities. However, the signal loss can be significant in the overfilled (irradiance) measurement mode. Again, high sensitivity is necessary to measure the decreased radiant flux on the detector in this measurement mode. In spite of the high performance lock-in amplifier used as a second stage signal-amplifier, the signal-to-noise ratio must be optimized for the output of the photocurrent measuring preamplifier. At low signal frequencies, the 1/*f* noise of HgCdTe detectors can be orders of magnitude larger than the noise from the background. It is important to keep the chopping frequency close to the elbow of the 1/*f* noise curve. As the elbow frequency depends on the bias voltage, biasing of PV detectors should be avoided in high sensitivity applications.

PV HgCdTe detectors can be used for optical radiation measurements from about 5 μm to 12 μm. At present, the optically sensitive diameter of these photodiodes is 2 mm or less. The shunt resistance of these devices is a few-hundred ohm and the capacitance is close to 1 nF for a 2 mm photodiode. The PV devices are linear only if the short-circuit current of the photodiode is measured. Photodiodes have smaller noise than PC detectors because the dominating photodiode shot-noise is much smaller than the generation-recombination noise of PC detectors. Bootstrapped preamplifiers have been reported [3, 4] that can keep the voltage drop on the photodiode very low resulting in an increased shunt resistance and better short circuit for the photodiode. The increased shunt resistance will result in smaller closed-loop voltage-gain and higher loop gain [5]. The smaller closed-loop voltage-gain produces lower voltage noise and voltage drift amplifications. The higher loop gain will decrease the uncertainty of the current-to-voltage conversion. Analysis and application of the bootstrap method for PV HgCdTe detectors is discussed in Sec. 3.

PC HgCdTe detectors can be used for longer wavelengths than PV detectors. We have used the PC HgCdTe detectors up to 20 μm, which is the long wavelength limit of the NIST IR spectral responsivity scale. PC detectors are operated with a dc bias current to sense the photogenerated conductance change, which is proportional to the radiant flux (power) on the detector. In a traditional biasing and measuring circuit, a dc source is connected to the detector through a load-resistor connected in series. The voltage change is measured across the detector while the high load-resistance keeps the bias current constant. It was shown in our earlier work [2] that the voltage measured on the detector is a nonlinear function of the conductance change. To eliminate this response non-linearity, a current measuring PC HgCdTe radiometer was developed [2]. This radiometer needed frequent zeroing to eliminate output voltage saturation caused by the non-stable dc compensation of the biasing source. The main concern of the new linear preamplifier design, described here, was to eliminate the need for output dc voltage compensations.

## 2. PC HgCdTe Detector and Preamplifier

Five PC HgCdTe detectors were tested. They had active areas of 3 mm × 3 mm or 4 mm × 4 mm. No cut-lines were fabricated in the detector centers to avoid large and sudden changes in the spatial response uniformity. In most commercial PC HgCdTe detectors, the detector segments, isolated by cutlines, are connected in series to increase the resultant resistance of the detector. The tested cutline-free detectors had very low resistance at 77 K, ranging from 15Ω to 22 Ω. A dc bias current of about 50 mA was needed for a detector to obtain a high enough detector responsivity. The test detector responsivities were very different. They ranged from a few V/W to 122 V/W when the broadband radiation of a ceramic glower was measured. The detector responsivity tests were made against pyroelectric detector standards of spectrally constant responsivity.

**Fig. 1:**
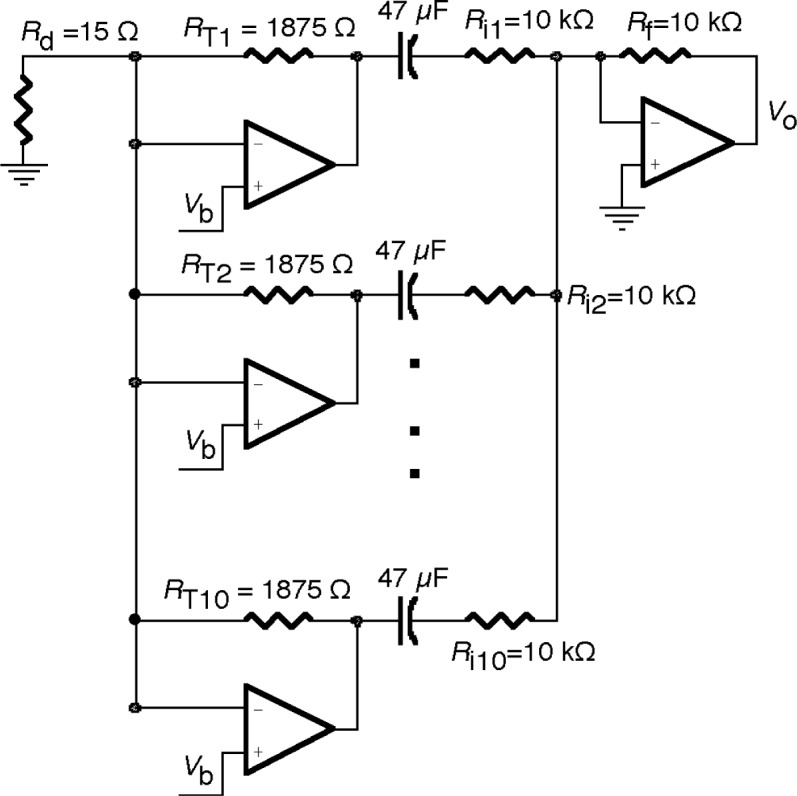
Multiple amplifier current-to-voltage converter for the PC HgCdTe detector.

The preamplifier was constructed from ten current-to-voltage converters connected in parallel as shown in the schematic of Fig. 1. Only 3 of the 10 amplifiers are shown in this drawing. The ten current-to-voltage converters produced 10 × 5 mA dc current to bias the PC detector. The voltage drop on the detector was determined by the bias voltage *V*_b_ connected to the non-inverting inputs of the operational amplifiers. The dc bias current requirement and the cold dc resistance of an individual detector determined the feedback resistor value for the operational amplifiers. The same feedback resistor value, *R*_1_ = *R*_2_ = *R*_10_ = 1875 Ω, was used in all of the ten parallel-connected amplifiers. The detector resistance *R*_d_ = 15 Ω corresponds to ten 150 Ω resistors connected in parallel, producing smaller feedback attenuation (from the operational amplifier output to its input) in each individual current-to-voltage converter. At low frequencies, the feedback network attenuation in each amplifier is 13.5 = (150 + 1875) Ω/150 Ω. The loop gain, which is the product of the feedback attenuation and the amplifier open-loop-gain, is close to 10^6^ for the applied AD797^1^ [6] amplifiers. Large loop gains result in high accuracy current-to-voltage conversion [5]. Note that each amplifier needs an offset equalizing resistor (not shown in the figure) connected in series to the non-inverting input. This resistor helps to keep the excess dc output voltage low. A value of 10Ω was used to compromise offset error versus gain error. The ac (10 Hz) signals from the ten channels of the preamplifier were combined with a sum-amplifier after the dc voltages at the outputs of the individual amplifiers were de-coupled. The low frequency roll-off point of the signal gain curve was tuned to 0.3 Hz by using serially connected 10 kΩ resistor and 47 μF capacitor for each channel.

As the conductance of the detector varies, there is a proportional variation in the output voltage of each preamplifier and in the variation of the summation amplifier. The conductance of the detector *G*_d_ varies only a small amount about the dc operating point. This incremental conductance change follows the rate of the radiation-chopper. As the measured change in the detector-current is proportional to the change of the detector conductance [2], the equivalent transfer resistance gain is found by taking the derivative of the output voltage *V*_o_ with respect to the detector conductance *G*_d_:

$$\partial V_{o}/\partial G_{d}=-V_{b}R_{f}R_{T}/R_{i}\;(V/S)$$ (1)

where

*V*_o_ is the output voltage in V,
*R*_f_ is the feedback resistor in the summing amplifier in Ω,
*R*_i_ is the input summing resistor of the summing amplifier in Ω,
*V*_b_ is the bias voltage in V,
*R*_T_ is the transfer (feedback) resistance of one of the input stages in Ω, and
*G*_d_ = 1/*R*_d_ is the conductance of the detector in S.

Because of the summing operation, the transfer resistance (current-to-voltage gain) is independent of the number of amplifiers used.

The advantage of this preamplifier design is that the dc output voltages of the individual operational amplifiers do not go into saturation and no compensating source is needed at the input. A compensating voltage source could produce large (and dominating) noise and also repeated compensations for zeroing the output voltage might be necessary.

From preamplifier output noise measurements (without the detector at the input) and the nominal 125 V/V preamplifier voltage-gain (calculated from Eq. (1) for a 15 Ω detector resistance), an input noise of 0.6 nV/Hz^1/2^ was obtained. This noise is comparable to the 0.5 nV/Hz^1/2^ Johnson noise of the 15 Ω detector resistance.

The 0.6 nV/Hz^1/2^ input voltage noise corresponds to a noise equivalent power (NEP) of 40 pW/Hz^1/2^ for a detector responsivity (without the preamplifier voltage-gain) of 16 V/W. This responsivity was measured on a typical test detector at 50 mA bias current and 10 Hz chopping frequency. The measured NEP was one order of magnitude larger, 0.34 nW/Hz^1/2^. The large noise was caused by the generation-recombination (G-R) noise of the biased PC HgCdTe detector. The calculated G-R current noise for 1 Hz bandwidth is:

$$I_{\text{GR}}={\left(2gqI_{d}\Delta f\right)}^{1/2}=0.57\;\text{nA}$$

where

*I*_d_ is the detector current, in our case 0.05 A,
*q* is the electron charge, 1.6 × 10^−19^ C,
*g* is the photoconductive gain (typically about 20 electrons per electron), and
Δ*f* is the electrical bandwidth in Hz.

Using the NEP = 0.34 nW/Hz^1/2^ and the detector area *A* = 0.16 cm^2^, a D* = *A*^1/2^/NEP = 10^9^ cm Hz^1/2^/W was calculated. The same detector was tested at a bias current of 10 mA where the detector responsivity decreased to 3.7 V/W.

At 50 mA bias, the resultant radiant power-to-voltage gain for the preamplifier output is 16 V/W × 125 V/V = 2000 V/W. The current-to-voltage gain is 1875 V/A. Accordingly, the power-to-current gain is about 1.1 A/W. From these gains and the measured 0.34 nW noise, a photoconductive gain of only 13 (from *I*_GR_ = 0.37 nA) is obtained.

For the NEP equal to 0.34 nW/Hz^1/2^, the output noise voltage of the preamplifier is 0.68 μV at a bandwidth of 1 Hz. The signal dynamic range, for a minimum signal-to-noise ratio of 100, will be from about 68 μV to 5 V (at the preamplifier output), which corresponds to a radiant power range of 34 nW to 2.5 mW. This is a dynamic range of close to five decades for radiant power measurements with 1 % (coverage factor *k* = 1) relative uncertainty.

## 3. PV HgCdTe Detector and Preamplifier

Instead of applying a bias voltage for the photodiode, we chose the bootstrap method to increase the shunt resistance of our PV HgCdTe detector. The shunt resistance increase was necessary to achieve high signal gains and to obtain low output noise for the preamplifier. The term bootstrapping refers to the use of an amplifier to increase the apparent impedance using near unity gain positive feedback [3,4]. Large area photon detectors may have relatively large capacitance and low shunt resistance. One method of dealing with this relatively low impedance is to use a near unity gain buffer amplifier to increase the effective equivalent impedance of the detector.

### 3.1 Bootstrap Impedance Multiplication Circuit

**Fig. 2:**
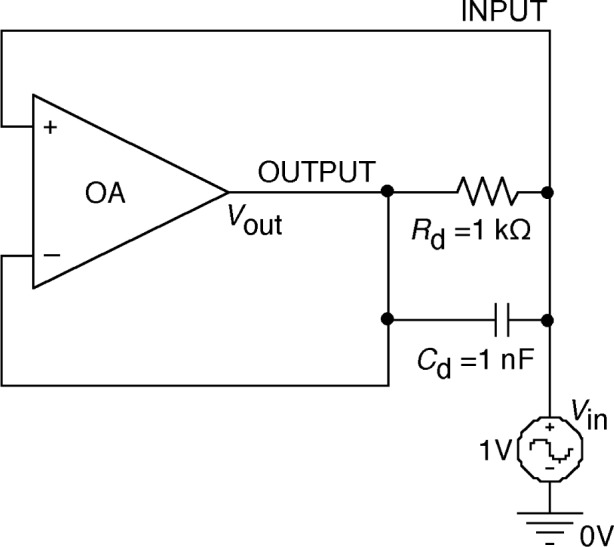
Computer simulation circuit to demonstrate the impedance multiplication concept.

The impedance multiplication circuit, using the unity gain buffer amplifier, is shown in Fig. 2. The unity gain buffer is implemented with an operational amplifier (OA). To demonstrate the impedance multiplication concept, a 1 V ac voltage source was connected to the input in a circuit simulation.

The effective equivalent input impedance which will be connected to the input of a second stage transfer resistance amplifier (not shown in Fig. 2) will be determined first. The input current can be calculated from Kirchoff’s and Ohm’s laws:

$$I_{\text{in}}(s)=Y_{d}[V_{\text{in}}(s)-V_{\text{out}}(s)]\;(A)$$ (2)

where

*Y*_d_ = *G*_d_ + *C*_d_s is the detector admittance,
*G*_d_ = 1/*R*_d_ is the conductance of the detector in S,
*C*_d_ is the junction capacitance of the detector in F,
*s* is angular frequency in the steady state frequency domain in rad/s; *s* = j2 π*f*,
*f* is frequency in Hertz, and j = (−1)^1/2^,
*I*_in_(*s*) is the input current in A,
*V*_in_(*s*) is the input voltage in V, and
*V*_out_(*s*) is the amplifier output voltage in V.

The closed loop voltage gain of the operational amplifier can be defined in terms of the open loop voltage gain as:

$$\frac{V_{\text{out}}(s)}{V_{\text{in}}(s)}=\frac{A(s)}{A(s)+1}\;(V/V)$$ (3)

where *A*(*s*) is the open loop gain of the operational amplifier in V/V.

The equivalent input impedance is defined as the ratio of the input voltage to the input current. Further, in order to eliminate the output voltage, Eq. (2) is written in the following form:

$$I_{\text{in}}(s)=V_{\text{in}}(s)[1-V_{\text{out}}(s)/V_{\text{in}}(s)]Y_{d}\;(A)$$ (4)

Inserting Eq. (3) into Eq. (4) becomes

$$I_{\text{in}}(s)=V_{\text{in}}(s)(1-A(s)/[A(s)+1)]Y_{d}\;(A)$$ (5)

Simplifying Eq. (5) and taking the voltage to current ratio, the equivalent impedance may be expressed as:

$$Z_{\text{in}}(s)=[A(s)+1]Z_{d}\;(\Omega )$$ (6)

where the detector impedance *Z*_d_ = 1/*Y*_d_.

Assume that the general form of *A*(*s*) may be expressed by a finite gain and a dominant pole [5], then:

$$A(s)=A_{o}/(\tau s+1)\;(V/V)$$ (7)

where

*A*_o_ is the dc open loop voltage gain in V/V, and
*τ* is the time constant of the dominant pole of the amplifier in s.

Inserting Eq. (7) into Eq. (6), the general form of the equivalent input impedance is written as follows:

$$Z_{\text{in}}(s)=\frac{R_{\text{in}}(1+s/\omega_{u})}{\left(\tau s+1)(R_{d}C_{d}s+1\right)}\;(\Omega )$$ (8)

where

*R*_in_ = *R*_d_(*A*_o_ + 1) in Ω,
*ω*_u_ = (*A*_o_ + 1)/*τ* in rad/s and is commonly called the unity gain bandwidth product for the operational amplifier.

**Fig. 3:**
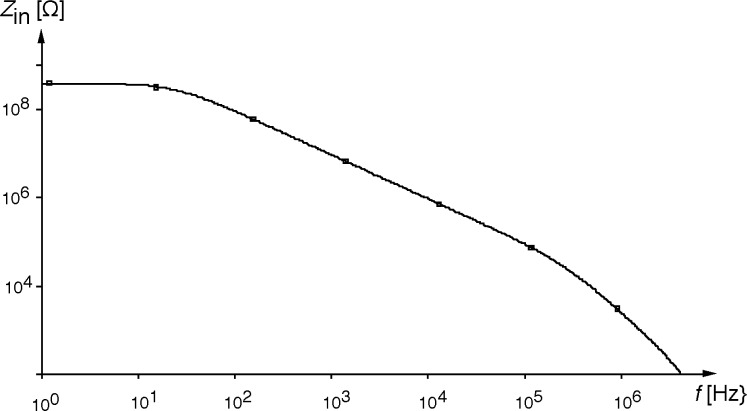
The equivalent input impedance calculated for a 1 kΩ shunt resistance and a 1 nF junction capacitance.

A typical operational amplifier would have an open loop gain of 400 V/mV and a dominant time constant of 16 ms. In the computer simulation, a 1 kΩ shunt resistance and a 1 nF capacitor along with a standard operational amplifier was used to demonstrate the multiplication of impedance concept. The simulation results are shown in Fig. 3.

For this example, at low frequencies the equivalent impedance is resistive and approaches 400 MΩ. There is a break point at 10 Hz and the impedance becomes capacitive over the band from 10 Hz to 1 MHz.

### 3.2 Bootstrap Transimpedance Amplifier, Signal Gain

**Fig. 4:**
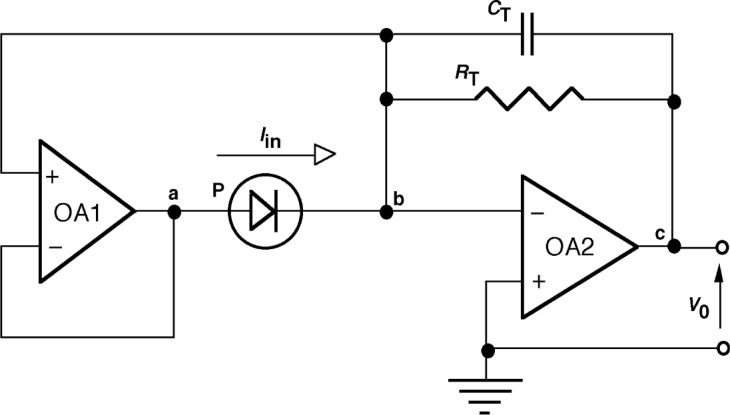
Bootstrap transimpedance amplifier.

**Fig. 5:**
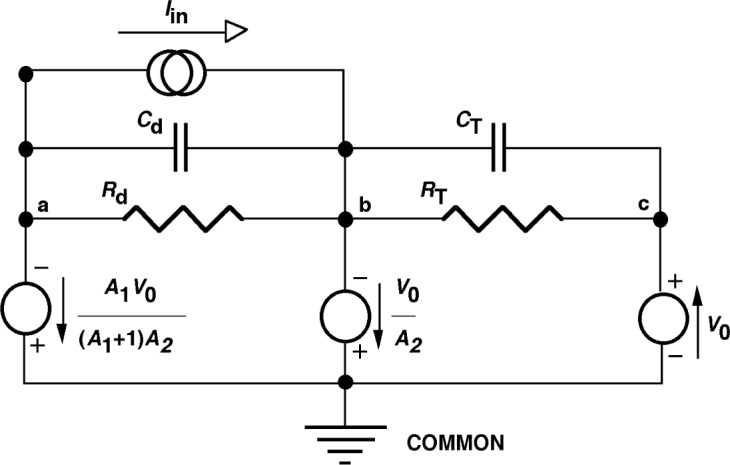
Three-node equivalent circuit of the combined transimpedance amplifier.

If we combine the bootstrap impedance multiplier circuit with a transimpedance amplifier, an improved performance current-to-voltage converter can be realized (Fig. 4). The feedback network of the second operational amplifier OA2 consists of a feedback capacitor *C*_T_ connected in parallel with the feedback resistor *R*_T_. The photocurrent *I*_in_ from the photodiode P is converted into an output voltage *V*_o_. The equivalent transfer function of the combined circuit has to be determined to obtain the signal gain. An equivalent circuit is shown in Fig. 5 for nodes a, b, and c.

The node-voltages [6], shown with voltage sources (circles), are referenced to the common and expressed from the output voltage *V*_o_. The voltage polarities are shown with both signs and arrows. The input voltage of the operational amplifier OA2 is on node b and it is equal to *V*_o_/*A*_2_. This voltage is multiplied by the gain of the buffer amplifier [shown by Eq. (3)]. The output voltage of the operational amplifier OA1 on node a will be *A*_1_*V*_o_/(*A*_1_ + 1)*A*_2_. The input current *I*_in_ is represented by a current source (double circle). It can be calculated from the node-voltage equation using Kirchoff’s current law [6] for node b:

$$I_{\text{in}}(s)=\left(\frac{V_{o}(s)A_{1}}{(A_{1}+1)A_{2}}-\frac{V_{o}(s)}{A_{2}}\right)\frac{1}{Z_{d}}-\left(\frac{V_{o}(s)}{A_{2}}+V_{o}(s)\right)\frac{1}{Z_{T}}\;(A)$$ (9)

Both *V*_0_(*s*) and *I*_in_(*s*) are frequency dependent. After simplifying and collecting terms, the output voltage to input current ratio (signal gain) can be written as:

$$\frac{V_{o}(s)}{I_{\text{in}}(s)}=\frac{-R_{T}}{1+sR_{T}C_{T}+\frac{1}{A_{2}}(1+sR_{T}C_{T}+\frac{R_{T}G_{d}+sR_{T}C_{d}}{A_{1}+1})}\;(\Omega )$$ (10)

where

*V*_o_(*s*) is the output voltage of OA2 in V,
*I*_in_(*s*) is the input current from the PV detector (photodiode) in A,
*A*_2_(*s*) is the open loop gain of OA2 in V/V,
*A*_1_(*s*) is the open loop gain of the bootstrap operational amplifier OA1 in V/V,
*R*_d_ is the shunt resistance of the photodiode, and
*C*_d_ is the junction capacitance of the photodiode.

**Fig. 6:**
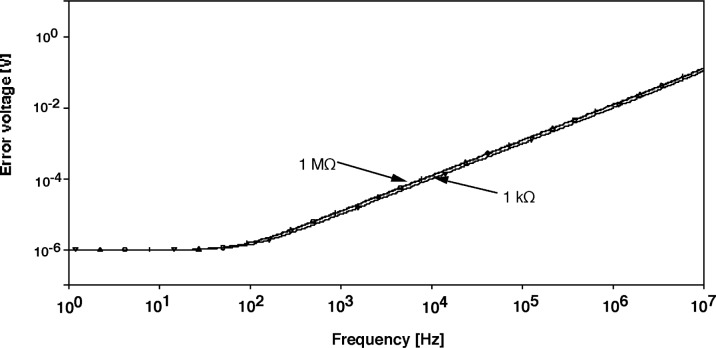
Error voltages (referenced to *V*_o_ = 1V) of the the bootstrap transimpedance amplifier for a shunt resistance of 200 Ω and feedback resistors of 1 kΩ to 1 MΩ.

**Fig. 7:**
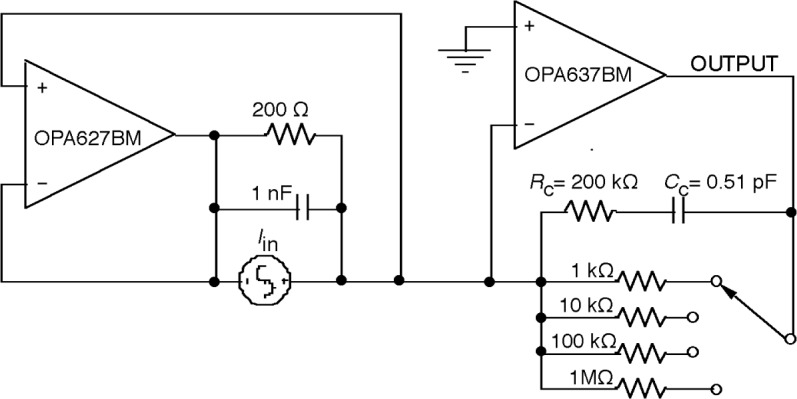
Computer simulation circuit for a bootstrap transfer resistance amplifier.

**Fig. 8:**
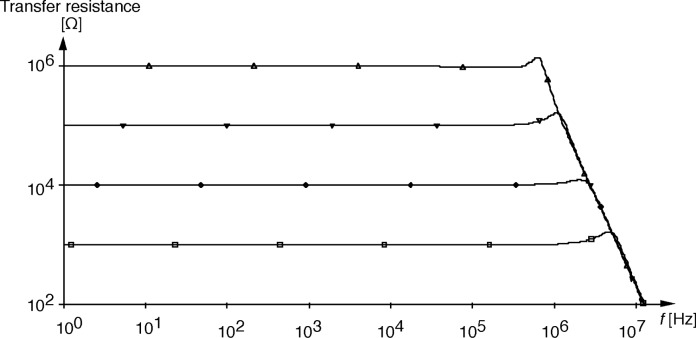
Transfer resistance (signal gain) of the bootstrap transimpedance amplifier with feedback resistor selections from 1 kΩ to 1 MΩ.

For high gain operational amplifiers, the transfer function in Eq. (10) becomes equal to −*R*_T_ which is the low frequency (dc) current-to-voltage gain of the combined transimpedance amplifier. The denominator describes the frequency dependent error of the photocurrent-to-voltage conversion. The error voltage curves of the signal conversion (gain) as calculated for the summing junction of the OA2 for an output signal of *V*_o_ = 1V are shown in Fig. 6. The combined circuit used in this computer simulation is shown in Fig. 7. The error voltage curves were calculated for a detector resistance of 200 Ω and feedback resistors from 1 kΩ to 100 MΩ. The transfer resistance curves, shown in Fig. 8, were calculated with feedback resistors up to 1 MΩ only.

The error voltage is the reciprocal of the loop gain which is, at low frequencies, roughly equal to the open loop gain (4 × 10^5^) of OA2. This is the result of the close to unity feedback attenuation at low frequencies, caused by the detector impedance multiplication.

The maximum signal current can be measured when the output signal is 1 V and the feedback resistance is 1 kΩ. This corresponds to a maximum photocurrent of 1 mA. The output signal maximum of 1 V is determined by the largest full-scale signal of the lock-in amplifier input where the output signal is connected. The relatively wide range of transfer resistance is one of the advantages of active impedance compensation. Note that *R*_c_ and *C*_c_ have been added to OA2 to provide voltage gain (gain peaking) compensation [5].

### 3.3 Output Noise

The output noise voltage as a power spectral density can be written as:

$${e_{\text{nout}}}^{2}=\frac{{\left[R_{T}G_{d}e_{n1}\times A_{1}/(A_{1}+1)\right]}^{2}+{\left\{e_{n2}[1+R_{T}G_{d}/(A_{1}+1)]\right\}}^{2}+4kTR_{T}+{\left(R_{T}i_{n1}\right)}^{2}+{\left(R_{T}i_{n2}\right)}^{2}+2qI_{d}{R_{T}}^{2}}{1+sR_{T}C_{T}+\frac{1}{A_{2}}\left(1+sR_{T}C_{T}+\frac{R_{T}G_{d}+sR_{T}C_{d}}{A_{1}+1}\right)}$$ (11)

where

*e*_nout_ is the equivalent output noise voltage density in V/Hz^1/2^,
*k* is the Boltzmann’s Constant (1.38 × 10^−23^ J/K),
*T* is the absolute temperature in K,
*e*_n1_ is the noise voltage density of the bootstrap amplifier in V/Hz^1/2^,
*e*_n2_ is the noise voltage density of the transfer resistance amplifier in V/Hz^1/2^,
*i*_n1_ is the equivalent input noise current density of the bootstrap amplifier in A/Hz^1/2^,
*i*_n2_ is the equivalent input noise current density of the transfer resistance amplifier in A/Hz^1/2^,
*I*_d_ is the average detector current and includes the dark leakage current in A, and
*q* is the electron charge equal to 1.602 × 10^−19^ Coulombs/electron.

The amplifier gains are taken at the frequency of interest and are in general complex. For low enough frequencies and high enough amplifier open loop gains, and where the input current noise of the operational amplifiers may be neglected, the denominator of Eq. (11) becomes unity and Eq. (11) simplifies to:

$${e_{\text{nout}}}^{2}={\left(R_{T}G_{d}e_{n1}\right)}^{2}+{\left[e_{n2}(1+G_{d}R_{T}/A_{1})\right]}^{2}+4kTR_{T}+2qI_{d}{R_{T}}^{2}\;{(V}^{2}/\mathrm{Hz})$$ (12)

In Eq. (12) the denominator error term of Eq. (11) drops out.

Equation (12) shows that the noise of the transfer resistance amplifier will in general be small compared to the voltage noise of the impedance feedback amplifier by virtue of the relatively large gain of this bootstrap operation. Clearly, for a large diode conductance, the noise of the bootstrap amplifier dominates. To avoid amplifier noise domination, the noise of the bootstrap amplifier’s voltage noise density must be less than or equal to the detector noise. Care should be taken because the 1/*f* amplifier voltage noise, at very low (dc) frequencies, can be much larger than the detector noise.

**Fig. 9:**
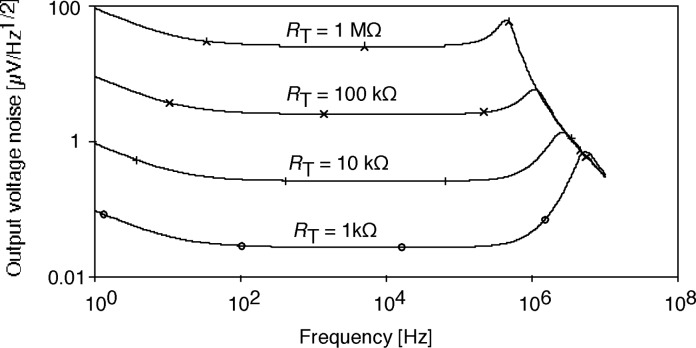
Output voltage noise density of the bootstrap transimpedance amplifier (shown in Fig. 7) for a detector shunt resistance of 200 Ω and various transfer resistance values.

The measured PV HgCdTe detector noise was 40 pA/Hz^1/2^ at 25 Hz. This shot-noise-dominated PV detector noise is about an order-of-magnitude smaller than the G-R-noise-dominated PC HgCdTe detector noise in Sec. 2. In order to match the amplifier noise to this detector noise, operational amplifiers OPA627 and 637 (as shown in Fig. 7) were used in the computer model. Figure 9 illustrates the output noise versus frequency characteristics of the bootstrap transimpedance amplifier for *R*_d_ = 200 Ω. The OA2 feedback resistors were selected in the simulator program between 1 kΩ and 1 MΩ in decade steps.

When the feedback resistor is increased to *R*_T_ = 100 MΩ the output voltage noise density will be close to 4 mV/Hz^1/2^. This corresponds to a minimum signal current of 40 pA which is equal to about 25 pW at a wavelength of 9 μm as calculated from the 1.6 A/W responsivity of the PV detector. With the described operational amplifier selection, the amplifier noise and the detector noise were equalized at the highest signal gain of 10^8^ V/A. For a measurement relative uncertainty of 1 % (*k* = 1) a signal range from about 4 nA to 1 mA could be achieved with the described component selections. As calculated from the 1.6 A/W responsivity, the 4 nA detector current corresponds to a radiant power of about 2.5 nW at the 9 μm peak responsivity of the 2 mm diameter PV HgCdTe detector.

### 3.4 Output Offset and Drift

The output offset voltage and its thermal drift is another concern if very large transfer resistance values are used. The output offset voltage, in terms of the input offset voltage, is:

$$V_{\text{outos}}=\frac{-R_{T}G_{d}V_{\text{os}1}\times A_{1}/(A_{1}+1)+V_{\text{os}2}(1+R_{T}G_{d}/(A_{1}+1))+(I_{B1}+I_{B2}+I_{\text{dark}})R_{T}}{1+sR_{T}C_{T}+\frac{1}{A_{2}}(1+sR_{T}C_{T}+\frac{R_{T}G_{d}+sR_{T}C_{d}}{A_{1}+1})}\;(V)$$ (13)

where

*V*_outos_ is the output offset voltage in V,
*V*_os1_ is the input offset voltage of the bootstrap amplifier in V,
*V*_os2_ is the input offset voltage of the transfer resistance amplifier in V,
*I*_B1_ is the input bias current of the bootstrap amplifier in A,
*I*_B2_ is the input bias current of the transfer resistance amplifier in A, and
*I*_dark_ is the dark current of the detector in A. Usually, the dark current of an unbiased photovoltaic detector is zero.

To evaluate a high offset voltage sensitivity case, we consider a 200 Ω detector with a 100 MΩ transfer resistance. An input offset voltage of only 1 μV can produce an output voltage of 0.5 V. This observation is counter-intuitive as both amplifiers provide equivalent open loop gain. In addition, a drift of 1 μV/K will produce a 0.5 V/K output drift. If such a system is desired for dc signal measurements then the amplifiers must be under temperature control and precise offset voltage trim has to be implemented. For ac (chopped radiation) measurements, the temperature control for the amplifiers may not be necessary but the offset trimming is still important to avoid saturation of the output voltage of the transfer resistance amplifier. Practical amplifiers are limited to one or two μV/K drift.

Care should be taken when high signal gains (transfer resistances) are selected because the very high feedback resistor and a very small detector shunt resistance can produce a very high closed loop voltage gain. The closed loop voltage gain, calculated as the inverse attenuation produced by the feedback network, cannot be larger than the open loop gain of the transfer resistance amplifier. From this restriction and the noise gain considerations in Sec. 3.3, the practical limit of the feedback resistor for a 200 Ω detector shunt resistance will be about 100 MΩ. This feedback network gives a closed-loop voltage gain of 5 × 10^5^, roughly equal to the open-loop voltage gain.

## 4. Conclusion

Because room temperature thermopiles and pyroelectric detectors do not have high enough sensitivities to measure weak signals from monochromators, HgCdTe detectors were selected as working standards for the 5 μm to 20 μm wavelength range. The high sensitivity and low uncertainty requirements for spectral responsivity measurements made it necessary to design linear and low noise photocurrent meters for both photoconductive (PC) and photovoltaic (PV) HgCdTe detectors. Using the results of the circuit analysis described here, high signal responsivities and low output noise can be achieved for the output of the preamplifiers even if very low detector resistances are applied.

PC detectors were used for the longer wavelength range where the detectors were biased with a dc current of 50 mA. A novel preamplifier was developed where the 50 mA bias was produced from ten current-to-voltage converters connected in parallel. The advantage of this preamplifier design was that the dc output voltages of the individual operational amplifiers did not go into saturation and no compensating voltage source was used at the input. Accordingly, repeated compensations were not needed and the dominating noise of the compensating source was eliminated. The input voltage noise of the parallel connected converters corresponded to an NEP of 40 pW/Hz^1/2^. The cold resistance of the cut-line free 3 mm by 3 mm detector was 15 Ω and the signal responsivity was 16 V/W. The measured NEP was an order of magnitude larger, dominated by the G-R noise of the current-biased PC detector. A radiant power range of 34 nW to 2.5 mW can be measured by this PC HgCdTe detector current meter with a relative standard uncertainty of 1 % (coverage factor *k* = 1).

Bootstrapping transimpedance amplifiers were designed and implemented to increase the equivalent impedance of PV HgCdTe detectors. The impedance multiplication concept has been analyzed and the equivalent input resistance has been computed versus frequency. For a detector shunt resistance of 1 kΩ and a junction capacitance of 1 nF, the equivalent impedance was resistive and approached 400 MΩ up to 10 Hz.

When this impedance multiplier was applied for a 200 Ω and 1 nF impedance of a PV HgCdTe detector, and it was combined with a transfer resistance amplifier, a range of transfer resistances from 1 kΩ to 100 MΩ could be implemented. The relatively wide range of transfer resistance is one of the advantages of active impedance compensation. The frequency dependent transimpedance (signal) gain of the bootstrap current-to-voltage converter was determined. The maximum 100 MΩ transfer resistance was dominated by the voltage gain for the input drift of the bootstrap amplifier. Also, this highest transfer resistance multiplied by the high detector conductance produced a high voltage gain for the input voltage noise of the bootstrap amplifier resulting in a noise equivalent photocurrent roughly equal to the PV HgCdTe detector noise current of 40 pA/Hz^1/2^. This noise current was measured on the PV HgCdTe detector used in our PV HgCdTe radiometer and it was about an order of magnitude smaller than the measured G-R noise of the current-biased PC HgCdTe detector. With a relative uncertainty of 1 % (*k* = 1), signals from 4 nA to 1 mA could be measured with the PV HgCdTe radiometer. The 4 nA detector current corresponds to a radiant power of 2.5 nW at the 9 μm peak responsivity (1.6 A/W) of the 2 mm diameter detector. The frequency dependent error voltage of the photocurrent-to-voltage conversion was determined.

The described frequency dependent gain equations and photocurrent measurement considerations can be applied to other PV and PC detectors as well, such as Ge, extended-InGaAs, InSb, and InAs photodiodes, and PbS or PbSe PC detectors.

## Footnotes

1 Certain commercial equipment, instruments, or materials are identified in this paper to foster understanding. Such identification does not imply recommendation or endorsement by the National Institute of Standards and Technology, nor does it imply that the materials or equipment identified are necessarily the best available for the purpose.
